# Case report: Spatiotemporal HER2 heterogeneity in AFP-producing gastric cancer: navigating long-term survival with molecularly-guided therapy in a refractory case

**DOI:** 10.3389/fimmu.2025.1696069

**Published:** 2026-01-02

**Authors:** Yanwen Diao, Haobo Yin, Xin Sun, Qian Dong, Jingdong Zhang

**Affiliations:** Medical Oncology Department of Gastrointestinal Tumors, Liaoning Cancer Hospital and Institute, Liaoning Key Laboratory of Gastrointestinal Cancer Translational Research, Cancer Hospital of China Medical University, Cancer Hospital of Dalian University of Technology, Shenyang, China

**Keywords:** heterogeneity, alpha-fetoprotein-producing gastric cancer, human epidermal growthfactor receptor 2, immunotherapy, anti-angiogenesis therapy

## Abstract

Alpha-fetoprotein-producing gastric cancer (AFPGC) is a rare, aggressive subtype with poor prognosis. We report a metastatic AFPGC case showing spatiotemporal human epidermal growth factor receptor 2 (HER2) heterogeneity. Following progression on first-line FLOT chemotherapy, the 65-year-old male received second-line apatinib plus programmed death-1 (PD-1) inhibitor. At progression (27 months), a lymph node biopsy revealed HER2 conversion from 1+ to 3 +. Third-line anti-HER2 antibody-drug conjugate (ADC) DP303c rapidly achieved partial response with normalized AFP. Despite treatment discontinuation due to neurotoxicity after 5 cycles, response persisted. Overall survival reached 79 months. This case highlights: 1) efficacy of anti-angiogenic and immunotherapy in AFPGC; 2) necessity of re-biopsy for detecting HER2 heterogeneity; and 3) potent activity of ADCs against HER2-converted metastases, enabling remarkable survival through sequential precision therapy.

## Introduction

Gastric cancer (GC) remains highly prevalent in China, with advanced disease exhibiting marked heterogeneity and poor prognosis, underscoring the need for molecular-level insights to guide treatment. AFPGC, a rare subtype representing 1.3%–15% of cases globally (1), is characterized by frequent liver metastasis, limited therapeutic options, and an unfavorable outlook. HER2 serves as a key therapeutic target in GC, though its heterogeneity—evidenced by discordance rates of 45%–79% by immunohistochemistry (IHC) and 23%–54% by *in situ* hybridization (ISH) (2), —complicates patient stratification and precise targeting. Here, we present a case of metastatic AFPGC with dynamic spatiotemporal HER2 heterogeneity, in which sequential molecularly-guided therapy enabled prolonged survival.

## Case presentation

**Figure 1:**
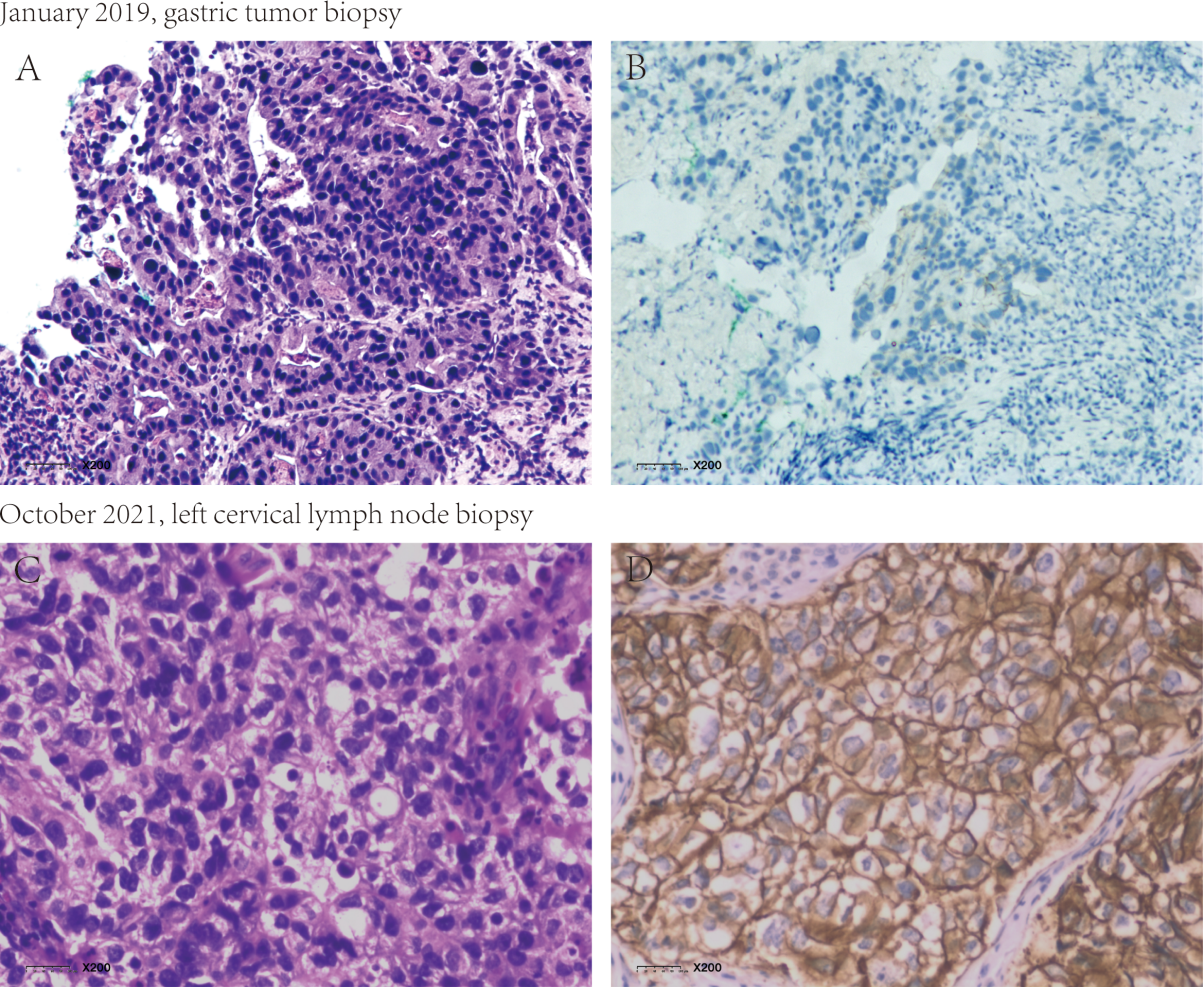
Histopathological and HER2 IHC analysis of the primary gastric tumor and metastatic lymph node. **(A, B)** HE staining and HER2 IHC of the primary gastric tumor biopsy, showing HER2 1+ (negative). **(C, D)** H&E staining and HER2 IHC of the metastatic left cervical lymph node biopsy, showing HER2 3+ (positive). HER2 IHC was performed using the Roche anti-HER2 rabbit monoclonal antibody (clone 4B5) and scored according to gastric cancer ASCO/CAP guidelines

**Figure 2:**
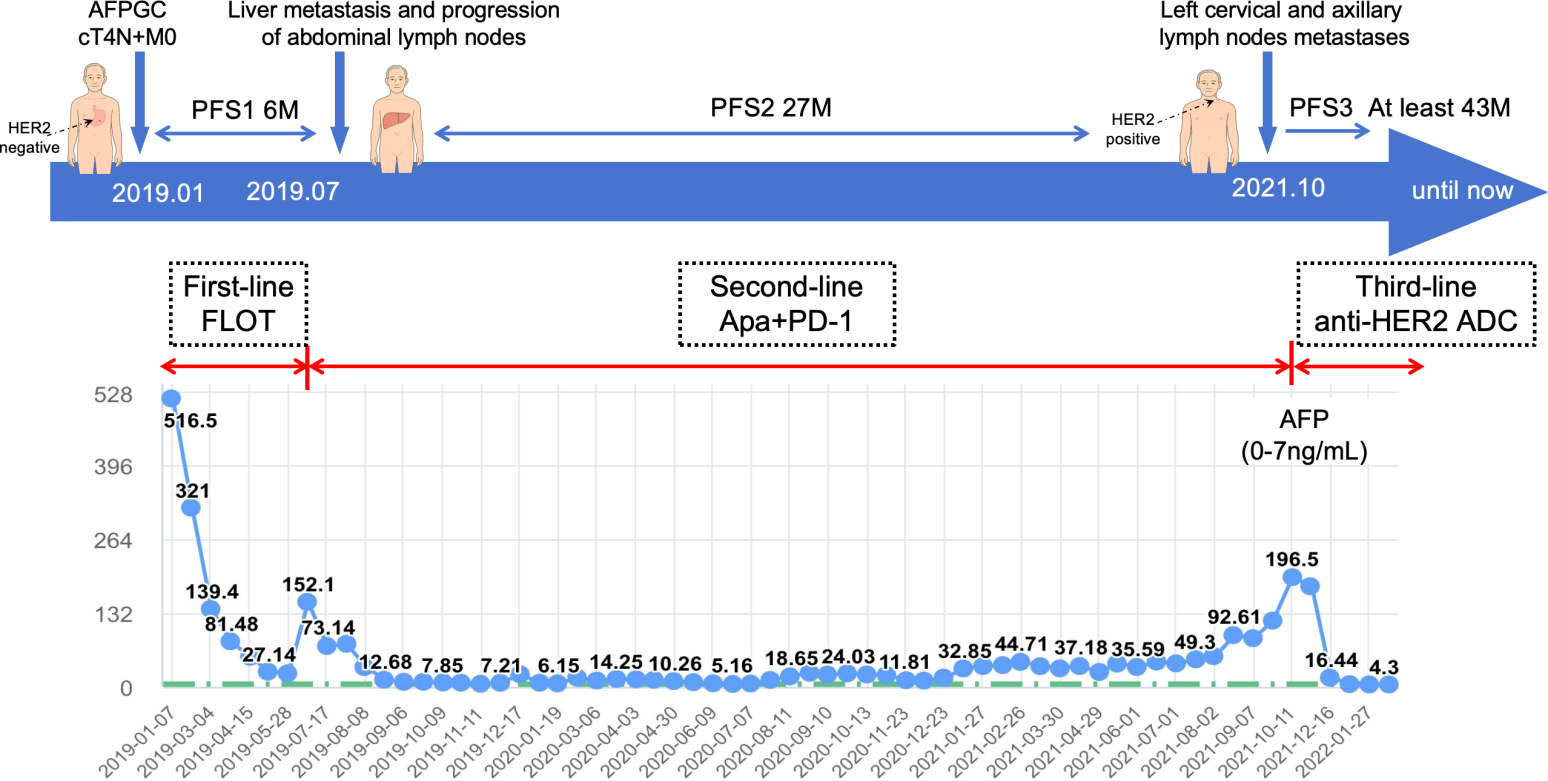
Timeline of the clinical course and AFP dynamics.

In January 2019, a 65-year-old male presenting with gastric discomfort underwent gastroscopy, which revealed a mass in the gastric body and antrum with histopathological confirmation of adenocarcinoma. IHC showed HER2 1+ (clone 4B5) and intact mismatch repair proteins (MSH2+, MSH6+, MLH1+, PMS2+) (**Supplementary Image 1**). Next-generation sequencing (NGS) indicated microsatellite stable (MSS) status and no ERBB2 amplification. Abdominal CT revealed gastric wall thickening and multiple enlarged lymph nodes in the porta hepatis and along the lesser curvature. The initial diagnosis was cT4aN+M0 gastric cancer. FLOT therapy (docetaxel, oxaliplatin, 5-fluorouracil) was started on January 24, 2019. After seven cycles, disease progressed with new liver metastases and lesser curvature lymph nodes enlargement, yielding a PFS of 6 months. Serum AFP was initially elevated at 516.5 ng/mL (normal: 0–7 ng/mL), declined to 27.14 ng/mL during therapy, but rebounded to 152.1 ng/mL at progression, confirming AFPGC. On July 25, 2019, the patient entered our IIT (ClinicalTrials.gov NCT04006821) receiving apatinib plus a PD-1 inhibitor as second-line therapy. PR was achieved after four cycles and sustained (**Supplementary Image 2**), with regression of liver and abdominal nodal lesions and normalized AFP. Adverse events included grade 3 proteinuria (apatinib-related), controlled by dose reduction, and grade 3 hypophysitis (PD-1 inhibitor-related), improved to grade 1 with hormone replacement.In October 2021, AFP rose to 195 ng/mL. CT showed progression in left cervical, supraclavicular, and axillary nodes, with stable abdominal and primary lesions. Gastroscopy showed no malignancy. Second-line PFS was 27 months. PET-CT confirmed metastatic lymph nodes; mild gastric uptake was considered post-treatment change. No metabolically active metastases were detected in the liver. A left cervical node biopsy (October 15, 2021) confirmed metastatic gastric adenocarcinoma. IHC showed HER2 3+ (clone 4B5), PD-L1 CPS <1 (Dako 22C3), Ki67 80%+, and the intact Mismatch repair protein. NGS (YucoOne^®^ Pro+ panel; depth 2134.4×; tumor content 55%) confirmed ERBB2 amplification (copy number 37.3), indicating HER2 heterogeneity from primary (1+) to metastasis (3+) (**Figure 1**). Beginning November 24, 2021, the patient received five cycles of DP303c, a novel anti-HER2 ADC, within a clinical trial (ClinicalTrials.gov NCT04826107). PR was observed after two cycles, coinciding with a rapid decline in serum AFP from 195 ng/mL to 16.44 ng/mL after the first cycle and subsequent normalization. Treatment was discontinued on April 2, 2022, following the fifth cycle due to grade 3 neurotoxicity, which manifested as persistent limb numbness, tingling, and restricted movement. Grade 2 ocular symptoms (decreased vision and dryness) also occurred. Supportive care, including neurotrophic agents, gabapentin, and traditional Chinese medicine acupuncture, led to gradual neurological improvement over approximately two years, while ocular symptomsgradually improved following the administration of artificial tears and steroid eye drops.Follow-up confirmed sustained PR, normalized AFP, no endoscopic evidence of disease, and an overall survival of 79 months (January 2019–August 2025) with a good quality of life. (**Figure 2**).

## Discussion

AFPGC is a rare variant, often misdiagnosed due to nonspecific clinical presentation. It is defined by elevated serum AFP (>20 ng/mL) or immunohistochemical evidence of AFP expression, and is associated with larger tumor size, frequent vascular and lymphatic invasion, and high liver metastasis rates (33%–72%) (3–5). AFP level serves as an independent prognostic factor, with 5-year survival declining sharply from 45.8% (AFP <20 ng/mL) to 7.7% (AFP >300 ng/mL) (6, 7). HER2 positivity is more common in AFPGC and hepatoid adenocarcinoma (21.8%–37.5%) than in conventional gastric cancer (12%–18%) (8, 9). The tumor microenvironment frequently shows VEGF-C overexpression and prominent angiogenesis (10), suggesting susceptibility to anti-angiogenic agents such as apatinib and ramuciruma (5, 11).

Despite limited evidence, immunotherapy shows promise in AFPGC. While Li et al. reported improved PFS with ICIs plus chemotherapy (22.0 vs. 4.3 months), PD-L1 CPS proved unreliable in predicting response (12). Furthermore, Liu et al. described a functionally heterogeneous CD8+ T-cell population co-expressing activation and exhaustion markers in hepatoid adenocarcinoma, affirming the immune context of these tumors (13). However, further exploration is needed to identify optimal combination strategies or more reliable predictive biomarke.

**Figure 3:**
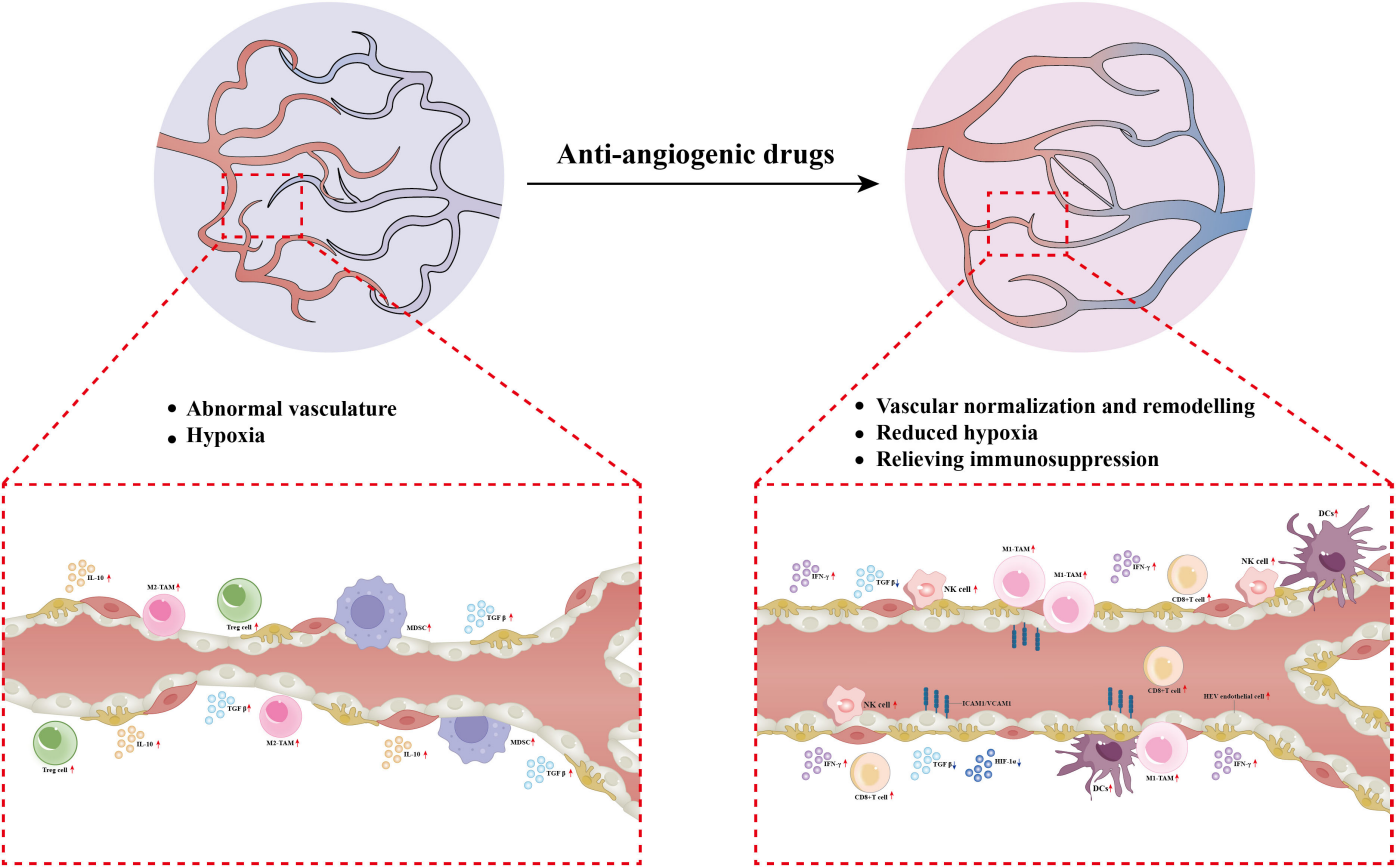
Proposed mechanism of synergistic effect between anti-angiogenic therapy and immunotherapy. Treg, Regulatory T Cell; MDSC, Myeloid-Derived Suppressor Cell; M2-TAM, M2-Polarized Tumor-Associated Macrophage; M1-TAM,M1-Polarized Tumor-Associated Macrophage; CD8^+^ T Cell, Cluster of Differentiation 8-positive T Lymphocyte; NK Cell, Natural Killer Cell; DCs, Dendritic Cell; HIF-1a, Hypoxia-Inducible Factor 1-alpha; TGF-b, Transforming Growth Factor-beta; IL-10, nterleukin-10;IFN-g, Interferon-gamma; ICAM-1, Intracellular Adhesion Molecule-1; VCAM-1, Vascular Cell Adhesion Molecule-1.

The combination of anti-angiogenic agents and immune checkpoint inhibitors(ICIs)has demonstrated considerable anti-tumor potential. Anti-angiogenic drugs targeting VEGF/VEGFR2 promote vascular normalization, alleviate hypoxia, and enhance the infiltration of CD8^+^ T cells (14, 15), and inducing high endothelial venules (HEVs) (16)—key components of tertiary lymphoid structures that support immune cell trafficking and activation. These agents further modulate the cytokine milieu by downregulating immunosuppressive factors such as HIF-1α and TGF-β, while enhancing the cytotoxicity of CD8^+^ T cells and NK cells (17–19). Additionally, they reduce recruitment of M2-type tumor-associated macrophages (TAMs) and regulatory T cells (Tregs), promote M1 polarization, and modulate immune-related genes such as ICAM-1, enhancing pro-inflammatory signaling including IFN-γ (20). These coordinated mechanisms enhance the depth and durability of immunotherapy responses (**Figure 3**). Given the promise of anti-VEGF and immunotherapy in AFPGC and its clinicopathological overlap with hepatocellular carcinoma, we initiated a clinical trial of apatinib plus a PD-1 inhibitor. This strategy provided a 27-month PFS in our second-line patient, remodeling the tumor immune microenvironment from an immune-excluded/desert to an immune-inflamed phenotype, thereby overcoming baseline PD-L1 negativity and microsatellite stability. Notably, the recent CAP 06 trial supports this strategy, reporting promising efficacy for a similar first-line regimen (21).

A striking feature of this case was the dynamic evolution of HER2 expression under therapeutic pressure. At progression, the patient developed extensive nodal metastases while other sites remained stable; a cervical node biopsy confirmed conversion from HER2-negative to HER2-positive status, revealing clear spatiotemporal heterogeneity.

HER2 heterogeneity in gastric cancer is well established, varying by subtype (higher in intestinal-type (22, 23) and showing 1%-14% primary-metastasis discordance (24). A meta-analysis reported 7% overall HER2 discordance, including 17% positive-to-negative and 4% negative-to-positive conversion (25). Mirroring this heterogeneity, 24%-35% of HER2-positive patients convert to negative post-trastuzumab (26), while studies like GASTHER1 (5.7%) (27) and GASTHER2 (4.0%) (28) demonstrate the reverse conversion. The clinical relevance is highlighted by a converted patient in GASTHER2 achieving 9.47 months PFS with T-DM1.

**Figure 4:**
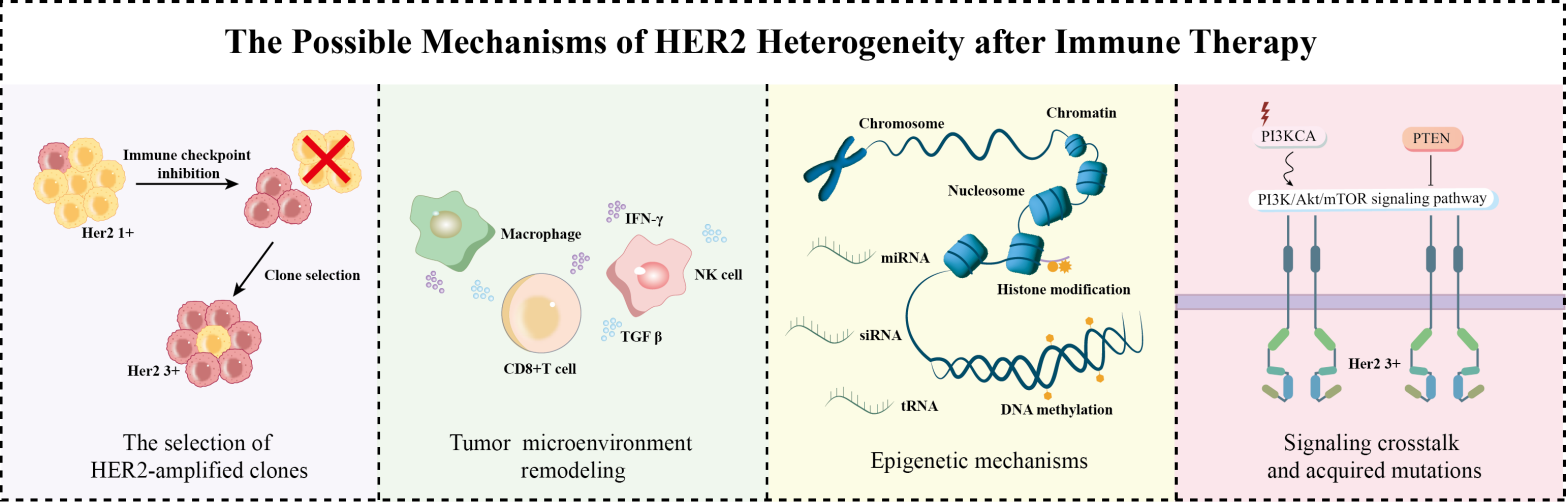
Proposed mechanisms underlying HER2 heterogeneity. PI3K, Phosphatidylinositol 3-kinase; Akt, Protein Kinase B; mTOR, Mammalian Target of Rapamycin; PTEN, Phosphatase and Tensin Homolog.

While the mechanisms underlying HER2 heterogeneity remain incompletely understood, sustained immune pressure may drive the selection of HER2-amplified clones.In this case, such pressure may have selected for HER2-amplified subclones with enhanced immune escape capabilities, progressively reshaping the overall HER2 expression profile (29). This process is further supported by observations in bladder urothelial carcinoma, where HER2 expression in recurrent tumors differs significantly from primary lesions (30, 31), underscoring the role of immune selection in modulating HER2 status (32). Concurrently, immune checkpoint inhibition remodels the tumor microenvironment, enabling cytokines—such as IFN-γ and TNF-α secreted by CD8^+^ T cells, NK cells, and macrophages—to exert dual regulatory effects on HER2 expression (33). In addition, potentially influencing HER2 expression via epigenetic mechanisms including promoter DNA methylation (34), histone modifications (35, 36), and non-coding RNA regulation (37, 38). Additional complexity arises from signaling crosstalk and acquired mutations in genes such as PIK3CA (39), collectively enhancing the plasticity of HER2 expression under immunotherapeutic pressure. Together, these mechanisms likely underlay the observed conversion of HER2 status in this patient following immunotherapy, as schematized in **Figure 4**.

This single-case report, while demonstrating encouraging outcomes, has inherent limitations that preclude broad generalizability. The survival benefit observed requires validation in larger prospective AFPGC cohorts. Additionally, the documented HER2 heterogeneity and clonal evolution, though supported by longitudinal biomarker assessment, warrant further investigation through multi-region sequencing or *in vitro* models. Notwithstanding these limitations, this case emphasizes the clinical value of repeat biopsy at progression to identify new targetable alterations such as HER2 conversion. Treatment with the novel anti-HER2 ADC DP303c resulted in manageable but clinically significant ocular and neurological toxicities, highlighting the need for vigilant monitoring and multidisciplinary management to maintain therapeutic continuity.

## Conclusions

The combination of anti-angiogenic agents and ICIs demonstrates efficacy in AFPGC. Our patient achieved sustained response with apatinib plus PD-1 inhibitor in the second-line setting, with manageable safety profile. Given gastric cancer’s heterogeneity, repeat biopsy upon progression proved crucial - revealing HER2 conversion that guided successful third-line anti-HER2 ADC therapy. This sequential precision approach enabled long-term survival in refractory metastatic AFPGC, highlighting the importance of dynamic biomarker-guided individualized treatment.

## Data Availability

The original contributions presented in the study are included in the article/**Supplementary Material**. Further inquiries can be directed to the corresponding authors.

## Supplementary material

Supplementary image 1 **(A, B)** Gastroscopy revealing stomach body and antrum occupied space. **(C)** HE revealed adenocarcinoma (×200 original magnification) **(D)** Immunohistochemistry revealed HER2(1+) (×200 original magnification).

Supplementary Image 2 Follow-up imaging demonstrated significant therapeutic response with apatinib plus PD-1 inhibitort.

Supplementary Image 3 PET-CT revealed hypermetabolic lymphadenopathy in the left cervical, supraclavicular, and axillary regions.

Supplementary Image 4 The NGS test indicated ERBB2 amplification (October 2021).

Supplementary Image 5 Following anti-HER2 ADC therapy, significant regression of metastatic lymph nodes was observed, meeting the criteria for PR.

## References

[1] McIntire KR Waldmann TA Moertel CG Go VL . Serum alpha-fetoprotein in patients with neoplasms of the gastrointestinal tract. Cancer Res. (1975) 35:991–6., PMID: 46783

[2] Wada R Hirabayashi K Ohike N Morii E . New guidelines for HER2 pathological diagnostics in gastric cancer. Pathol Int. (2016) 66:57–62. doi: 10.1111/pin.12390, PMID: 26814046

[3] Luo B Wu Z Hu C Xie W He J Liu H . Alpha fetoprotein (AFP)-producing gastric cancer: clinicopathological features and treatment strategies. Cell bioscience. (2025) 15:82. doi: 10.1186/s13578-025-01424-8, PMID: 40495217 PMC12153134

[4] Hirajima S Komatsu S Ichikawa D Kubota T Okamoto K Shiozaki A . Liver metastasis is the only independent prognostic factor in AFP-producing gastric cancer. World J gastroenterol. (2013) 19:6055–61. doi: 10.3748/wjg.v19.i36.6055, PMID: 24106406 PMC3785627

[5] Li N Bai C Zhang R Ma L Ren X Zhang J . Efficacy and safety of apatinib for the treatment of AFP-producing gastric cancer. Trans Oncol. (2021) 14:101004. doi: 10.1016/j.tranon.2020.101004, PMID: 33383486 PMC7777135

[6] Lin HJ Hsieh YH Fang WL Huang KH Li AF . Clinical manifestations in patients with alpha-fetoprotein-producing gastric cancer. Curr Oncol (Toronto Ont). (2014) 21:e394–399. doi: 10.3747/co.21.1768, PMID: 24940098 PMC4059802

[7] Zhan Z Chen B Yu J Zheng J Zeng Y Sun M . Elevated serum alpha-fetoprotein is a significant prognostic factor for patients with gastric cancer: results based on a large-scale retrospective study. Front Oncol. (2022) 12:901061. doi: 10.3389/fonc.2022.901061, PMID: 35847953 PMC9277009

[8] He F Fu Y Sun Q Geng P Zheng Z Pu X . Integrated clinicopathological and immunohistochemical analysis of gastric adenocarcinoma with hepatoid differentiation: an exploration of histogenesis, molecular characteristics, and prognostic markers. Hum pathol. (2021) 115:37–46. doi: 10.1016/j.humpath.2021.02.003, PMID: 33636206

[9] Akazawa Y Saito T Hayashi T Yanai Y Tsuyama S Akaike K . Next-generation sequencing analysis for gastric adenocarcinoma with enteroblastic differentiation: emphasis on the relationship with hepatoid adenocarcinoma. Hum pathol. (2018) 78:79–88. doi: 10.1016/j.humpath.2018.04.022, PMID: 29751042

[10] Kamei S Kono K Amemiya H Takahashi A Sugai H Ichihara F . Evaluation of VEGF and VEGF-C expression in gastric cancer cells producing alpha-fetoprotein. J gastroenterol. (2003) 38:540–7. doi: 10.1007/s00535-002-1099-y, PMID: 12825129

[11] Zhu XR Zhu ML Wang Q Xue WJ Wang YW Wang RF . A case report of targeted therapy with apatinib in a patient with advanced gastric cancer and high serum level of alpha-fetoprotein. Medicine. (2016) 95:e4610. doi: 10.1097/md.0000000000004610, PMID: 27631210 PMC5402553

[12] Li W Li Q Yu Y Wang Y Chen E Chen L . Effect of immune checkpoint inhibitors plus chemotherapy on advanced gastric cancer patients with elevated serum AFP or hepatoid adenocarcinoma. Cancer Manage Res. (2020) 12:11113–9. doi: 10.2147/cmar.s276969, PMID: 33173344 PMC7646478

[13] Liu Z Wang A Pu Y Li Z Xue R Zhang C . Genomic and transcriptomic profiling of hepatoid adenocarcinoma of the stomach. Oncogene. (2021) 40:5705–17. doi: 10.1038/s41388-021-01976-2, PMID: 34326469

[14] Xu Q Shao D . Leveraging the synergy between anti-angiogenic therapy and immune checkpoint inhibitors to treat digestive system cancers. Front Immunol. (2024) 15:3389/fimmu.2024.1487610. doi: 10.3389/fimmu.2024.1487610, PMID: 39691707 PMC11649667

[15] Lin QX Song WW Xie WX Deng YT Gong YN Liu YR . Sequential treatment of anti-PD-L1 therapy prior to anti-VEGFR2 therapy contributes to more significant clinical benefits in non-small cell lung cancer. Neoplasia (New York NY). (2025) 59:101077. doi: 10.1016/j.neo.2024.101077, PMID: 39561585 PMC11617296

[16] Kabir AU Subramanian M Kwon Y Choi K . Linking tumour angiogenesis and tumour immunity. Nat Rev Immunol. (2025) 14. doi: 10.1038/s41577-025-01211-z, PMID: 40813802

[17] Li X Luo X Chen S Chen J Deng X Zhong J . All-trans-retinoic acid inhibits hepatocellular carcinoma progression by targeting myeloid-derived suppressor cells and inhibiting angiogenesis. Int immunopharmacol. (2023) 121:110413. doi: 10.1016/j.intimp.2023.110413, PMID: 37301119

[18] Li BB Jiang YY Li X Yu MM Meng Q Wang DN . Qingrehuoxue formula enhances anti-PD-1 immunotherapy in NSCLC by remodeling the tumor immune microenvironment via TREM2 signaling. BMC complement Med therapies. (2025) 25:270. doi: 10.1186/s12906-025-05020-8, PMID: 40670983 PMC12269164

[19] Zhang Q Liu H Wang H Lu M Miao Y Ding J . Lenvatinib promotes antitumor immunity by enhancing the tumor infiltration and activation of NK cells. Am J Cancer Res. (2019) 9:1382–95., PMID: 31392076 PMC6682710

[20] Zhou Y Ren D Bi H Bing Y Cai Z Hong W . Tumor-associated macrophage: Emerging targets for modulating the tumor microenvironment. Zhongguo fei ai za zhi = Chin J Lung cancer. (2024) 27:231–40. doi: 10.3779/j.issn.1009-3419.2024.102.13, PMID: 38590197 PMC11002190

[21] Wang Y Lu J Chong X Wang C Chen X Peng Z . PD-1 antibody camrelizumab plus apatinib and SOX as first-line treatment in patients with AFP-producing gastric or gastro-esophageal junction adenocarcinoma (CAP 06): a multi-center, single-arm, phase 2 trial. Signal transduct targeted Ther. (2025) 10:100. doi: 10.1038/s41392-025-02193-z, PMID: 40082418 PMC11906745

[22] Ge X Wang H Zeng H Jin X Sujie A Xu C . Clinical significance of assessing Her2/neu expression in gastric cancer with dual tumor tissue paraffin blocks. Hum pathol. (2015) 46:850–7. doi: 10.1016/j.humpath.2015.02.011, PMID: 25863425

[23] He C Bian XY Ni XZ Shen DP Shen YY Liu H . Correlation of human epidermal growth factor receptor 2 expression with clinicopathological characteristics and prognosis in gastric cancer. World J gastroenterol. (2013) 19:2171–8. doi: 10.3748/wjg.v19.i14.2171, PMID: 23599643 PMC3627881

[24] Wei SY Xiao H Zheng HX . Research progress of HER2 heterogeneity in gastric cancer. J Clin Pathol Res. (2019) 39:2014–20. doi: 10.3978/j.issn.2095-6959.2019.09.026

[25] Peng Z Zou J Zhang X Ye Y Jing G Yi L . HER2 discordance between paired primary gastric cancer and metastasis: a meta-analysis. Chin J Cancer Res = Chung-kuo yen cheng yen chiu. (2015) 27:163–71. doi: 10.3978/j.issn.1000-9604.2014.12.09, PMID: 25937778 PMC4409975

[26] Valenza C Guidi L Battaiotto E Trapani D Sartore Bianchi A Siena S . Targeting HER2 heterogeneity in breast and gastrointestinal cancers. Trends cancer. (2024) 10:113–23. doi: 10.1016/j.trecan.2023.11.001, PMID: 38008666

[27] Park SR Park YS Ryu MH Ryoo BY Woo CG Jung HY . Extra-gain of HER2-positive cases through HER2 reassessment in primary and metastatic sites in advanced gastric cancer with initially HER2-negative primary tumours: Results of GASTric cancer HER2 reassessment study 1 (GASTHER1). Eur J Cancer (Oxford England: 1990). (2016) 53:42–50. doi: 10.1016/j.ejca.2015.09.018, PMID: 26693898

[28] Hyung J Kim HD Ryu MH Park YS Moon M Kang YK . GASTric cancer HER2 re-assessment study 2 (GASTHER2): HER2 re-assessment for initially HER2-negative advanced gastric cancer patients after progression on first-line treatment. Cancer Res Treat. (2024) 56:199–207. doi: 10.4143/crt.2023.490, PMID: 37340843 PMC10789948

[29] Yang Y Sun Q Deng Z Shi W Cheng H . Cbl induced ubiquitination of HER2 mediate immune escape from HER2-targeted CAR-T. J Biochem Mol toxicol. (2023) 37:e23446. doi: 10.1002/jbt.23446, PMID: 37354072

[30] Yang M Yao Y Wang K Qi L Yang B Khudadad M . Clinicopathological characteristics and prognostic significance of HER2 status evaluation in patients with urothelial carcinoma: a retrospective single-center experience in China. Virchows Archiv: an Int J pathol. (2025) 26. doi: 10.1007/s00428-025-04057-x, PMID: 40011272

[31] Ali MY Aboelsaad AY Abdel Gawad AM Abouelgreed TA El Gammal AA Ghoneimy OM . HER2/neu expression status of post BCG recurrent non-muscle-invasive bladder urothelial carcinomas in relation to their primary ones. Archivio italiano di urologia andrologia: organo ufficiale [di] Societa italiana di ecografia urologica e nefrologica. (2023) 95:11313. doi: 10.4081/aiua.2023.11313, PMID: 37254927

[32] Matusz-Fisher A Tan AR . Combination of HER2-targeted agents with immune checkpoint inhibitors in the treatment of HER2-positive breast cancer. Expert Opin Biol Ther. (2022) 22:385–95. doi: 10.1080/14712598.2021.1981284, PMID: 34806498

[33] Cheng Y Li Q Kong Y Huang A Yang Z Ying T . An IL-15-modified NKp30×HER2 trispecific NK cell engager enhances NK cell activation and tumor cell killing. J leukocyte Biol. (2025) 117:qiaf107. doi: 10.1093/jleuko/qiaf107, PMID: 40694640

[34] Nami B Ghanaeian A Black C Wang Z . Epigenetic silencing of HER2 expression during epithelial-mesenchymal transition leads to trastuzumab resistance in breast cancer. Life (Basel Switzerland). (2021) 11:868. doi: 10.3390/life11090868, PMID: 34575017 PMC8472246

[35] Liu Q Borcherding NC Shao P Maina PK Zhang W Qi HH . Contribution of synergism between PHF8 and HER2 signalling to breast cancer development and drug resistance. EBioMedicine. (2020) 51:102612. doi: 10.1016/j.ebiom.2019.102612, PMID: 31923801 PMC7000350

[36] Wegwitz F Prokakis E Pejkovska A Kosinsky RL Glatzel M Pantel K . The histone H2B ubiquitin ligase RNF40 is required for HER2-driven mammary tumorigenesis. Cell Death dis. (2020) 11:873. doi: 10.1038/s41419-020-03081-w, PMID: 33070155 PMC7568723

[37] Tian D Tian M Ma ZM Zhang LL Cui YF Li JL . Anesthetic propofol epigenetically regulates breast cancer trastuzumab resistance through IL-6/miR-149-5p axis. Sci Rep. (2020) 10:8858. doi: 10.1038/s41598-020-65649-y, PMID: 32483313 PMC7264192

[38] Zhao H Hu H Li Z Xu M Miao P Chen B . The mechanism of ncRNA in trastuzumab resistance in HER2-positive tumors. Med Oncol (Northwood London England). (2025) 42:415. doi: 10.1007/s12032-025-02976-y, PMID: 40779123

[39] Lee K Lee J Choi J Sim SH Kim JE Kim MH . Genomic analysis of plasma circulating tumor DNA in patients with heavily pretreated HER2 + metastatic breast cancer. Sci Rep. (2023) 13:9928. doi: 10.1038/s41598-023-35925-8, PMID: 37336919 PMC10279711

