# Supplementary figures and images for: Case report: Spatiotemporal HER2 heterogeneity in AFP-producing gastric cancer: navigating long-term survival with molecularly-guided therapy in a refractory case
Source: Front Immunol. 2026 Jan 2;16:1696069. doi: 10.3389/fimmu.2025.1696069 (PMC12808426; doi:10.3389/fimmu.2025.1696069)

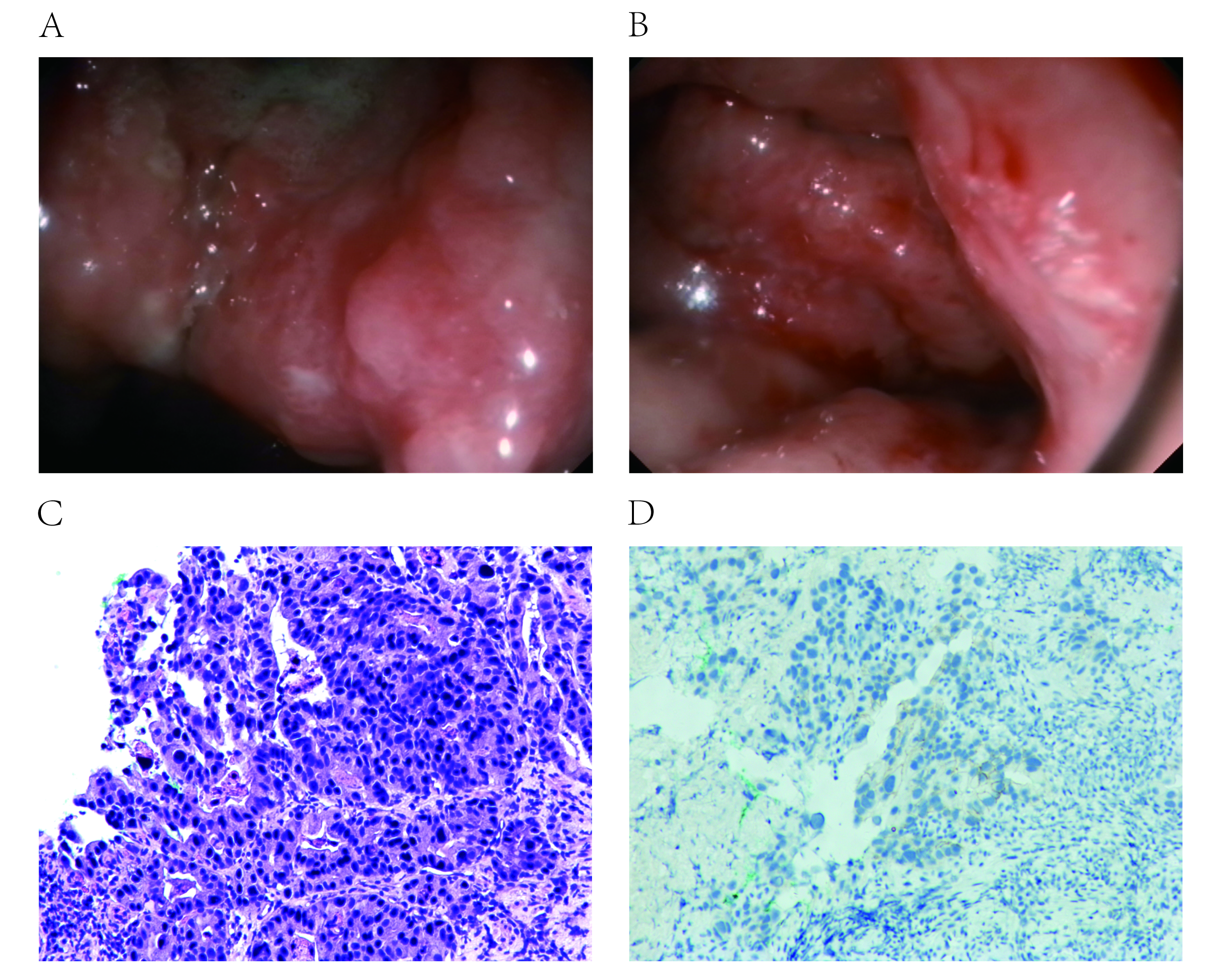

Supplement: Supplementary image 1 — (A, B) Gastroscopy revealing stomach body and antrum occupied space. (C) HE revealed adenocarcinoma (×200 original magnification) (D) Immunohistochemistry revealed HER2(1+) (×200 original magnification). [file Image1.tif]

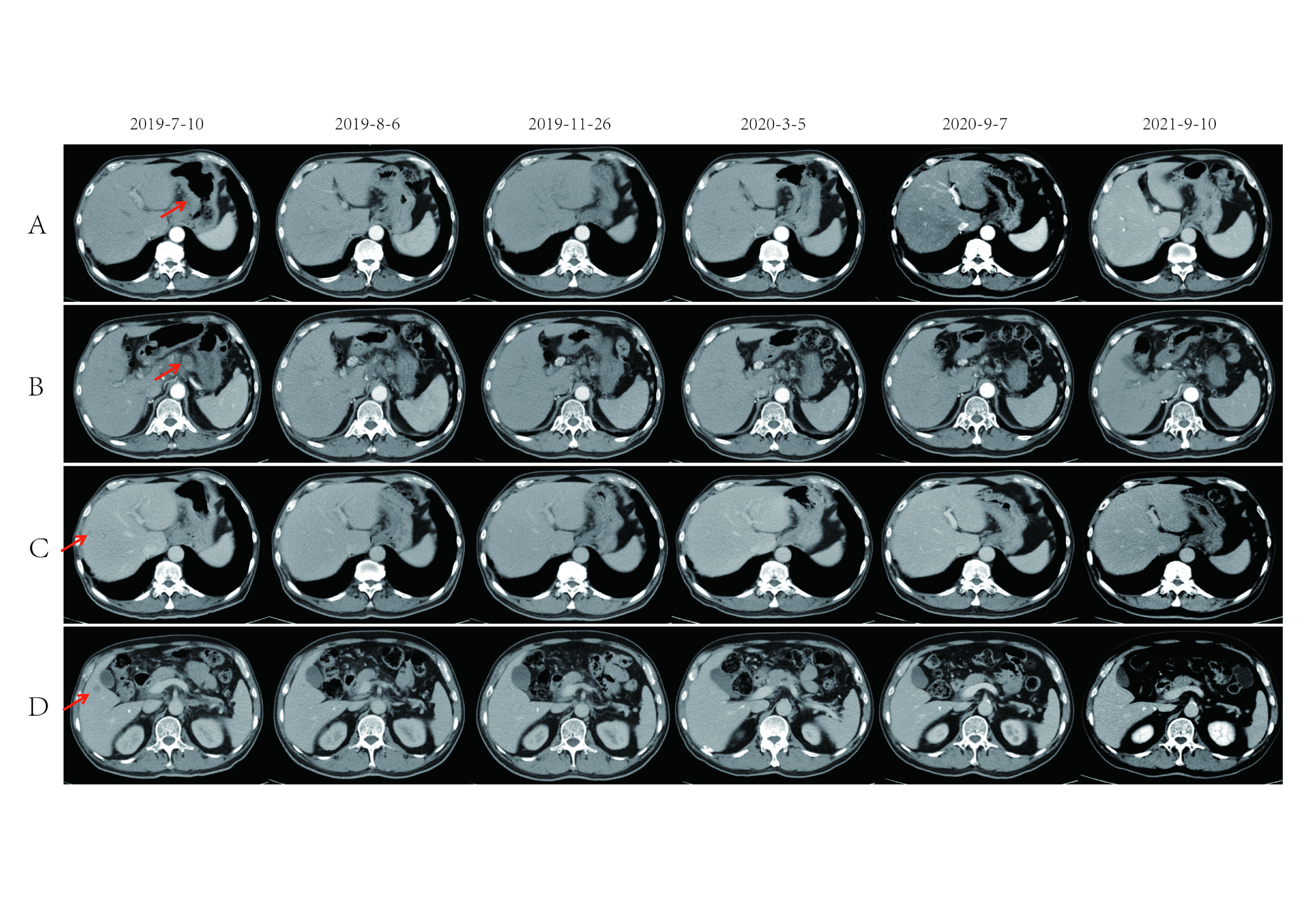

Supplement: Supplementary Image 2 — Follow-up imaging demonstrated significant therapeutic response with apatinib plus PD-1 inhibitort. [file Image2.tif]

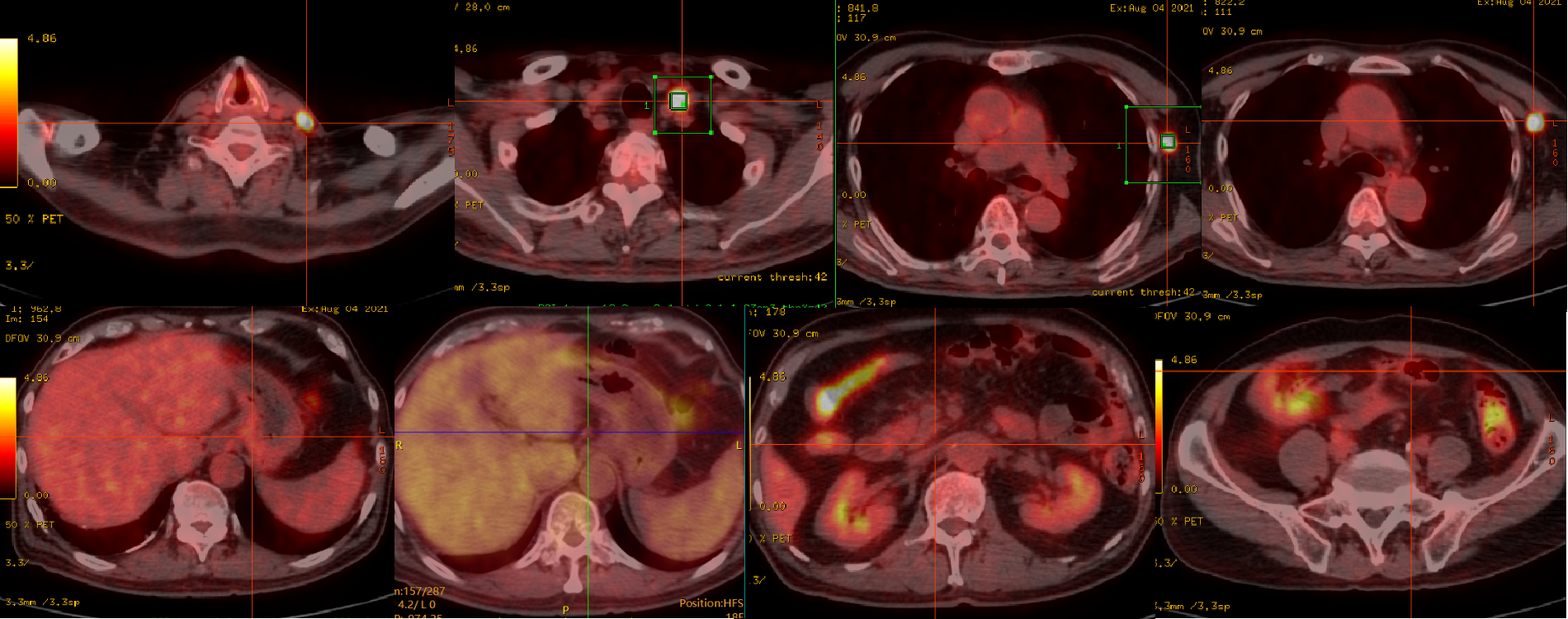

Supplement: Supplementary Image 3 — PET-CT revealed hypermetabolic lymphadenopathy in the left cervical, supraclavicular, and axillary regions. [file Image3.tif]

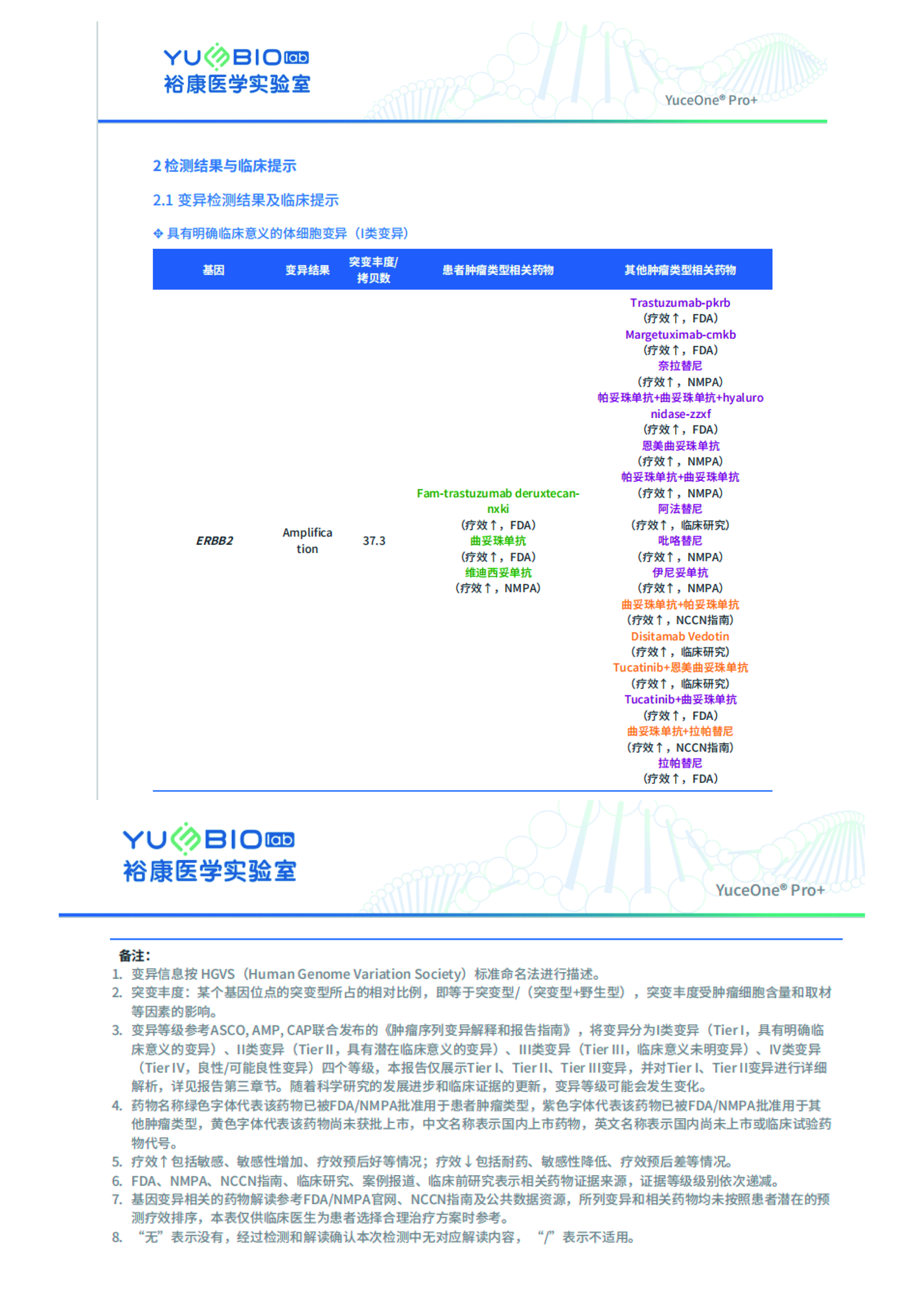

Supplement: Supplementary Image 4 — The NGS test indicated ERBB2 amplification (October 2021). [file Image4.tif]

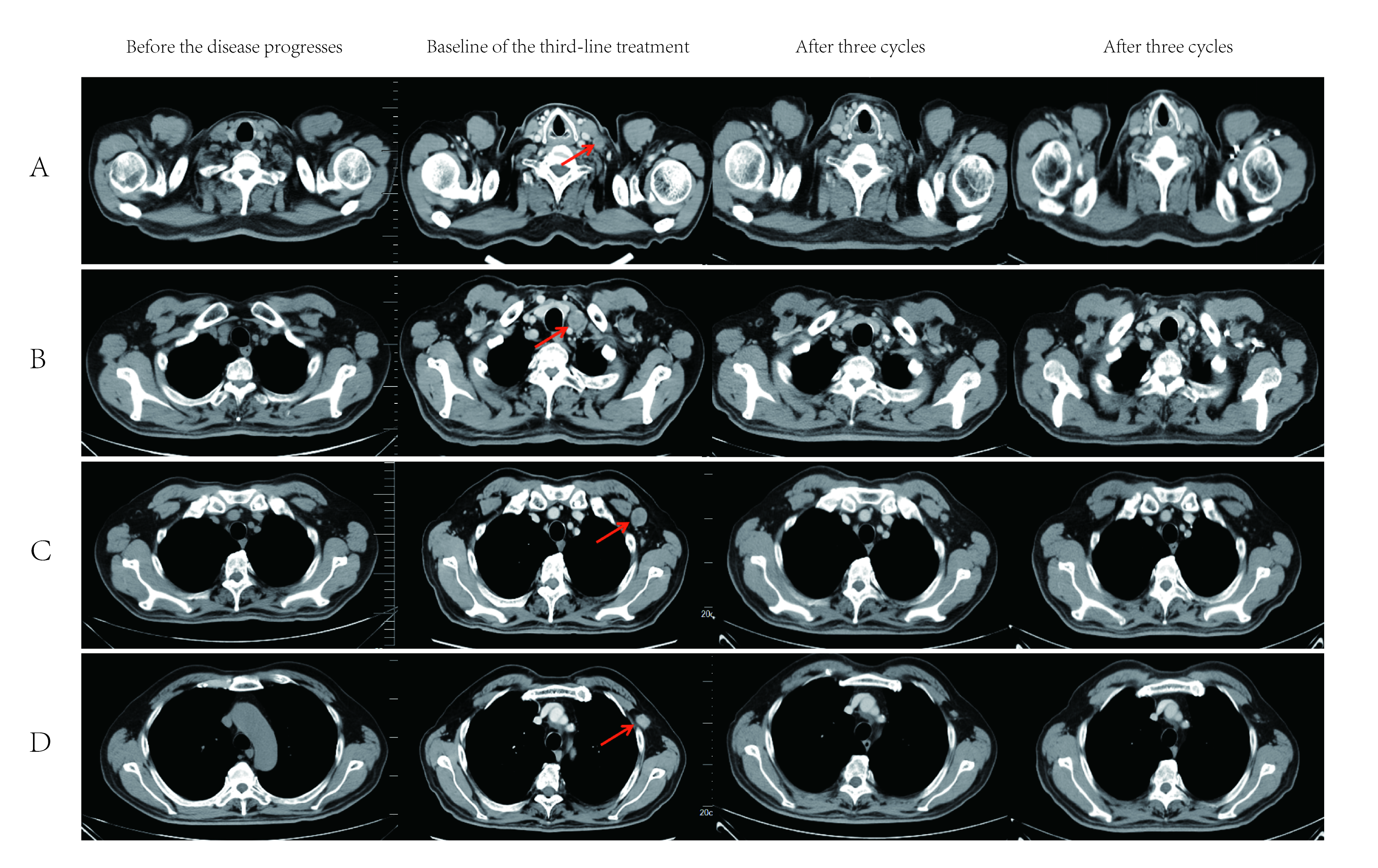

Supplement: Supplementary Image 5 — Following anti-HER2 ADC therapy, significant regression of metastatic lymph nodes was observed, meeting the criteria for PR. [file Image5.tif]
